# The Morality of Vivisection

**Published:** 1892-11-05

**Authors:** Victor Horsley


### The Morality of Vivisection.

By Professor Victor Horsley, F.R.S.

[In response to our request Professor Victor Horsley has furnished us with the following notes.]

The Church is really at one with medicine and science. This was clearly shown at the Congress by the attitude of the clergy there. My aim at the Church Congress was to point out to the public—

(*a*) That the only persons capable of appreciating properly the value of experiments on animals are those who have honestly consulted and studied the text books of physiology, pathology, medicine, and surgery.

(*b*) That the medical profession, in Congress, has absolutely and without a single dissentient voice expressed itself keenly appreciative of the value of "Vivisection."

(*c*) That this expression ought to be loyally accepted, especially by those who constantly appeal to the profession for help.

(*d*) That Mr. Lawson Tait and the other half dozen have never dared to publicly—in medical gatherings—attack the above resolutions unanimously passed, and that therefore they ought not to be heard with credence.

(*e*) That the two Bishops, Dr. Barry and Dr. Moorhouse, have acted in opposition to all moral precedent in violently attacking, as they have done since 1891, the medical profession, without the least inquiry into the truth of the basis of their action.

(*f*) That that basis consists of nothing but the irresponsible statements of Miss Cobbe.

(*g*) That Miss Cobbe, as shown by documents published and signed by her, has, ever since this agitation begun sixteen years or more ago, persistently falsified the truth.

Of these, by way of example, see *Times* (Tuesday, October 25th).

